# Land Use and Land Cover Dynamics and Properties of Soils under Different Land Uses in the Tejibara Watershed, Ethiopia

**DOI:** 10.1155/2020/1479460

**Published:** 2020-09-01

**Authors:** Sabiela Fekad Fentie, Kehali Jembere, Endalkachew Fekadu, Dessale Wasie

**Affiliations:** ^1^Department of Natural Resources Management, University of Gondar, P.O. Box 196, Gondar, Ethiopia; ^2^Department of Soil and Water Resources Management, Woldia University, P.O. Box 400, Woldia, Ethiopia

## Abstract

Land use changes have long been considered among many factors responsible for physical and chemical soil degradation. This study was conducted to evaluate land use and land cover (LULC) changes and their cumulative effects over 30 years (from 1989 to 2019) on the current physical and chemical properties of soils in the Tejibara watershed, Ethiopia. Image analysis and LULC classifications were performed using ERDAS IMAGINE 2014 and ArcGIS 10.4 software, respectively. For the determination of soil properties, four land use types (natural forest, eucalyptus plantation, cultivated, and grazing lands) and two soil depths (0–20 and 20–40 cm) were used. Triplicate composite soil samples were collected from each land use type and soil depths. For the determination of physical (texture and bulk density) and chemical soil properties such as electrical conductivity (EC), organic matter (OM), total nitrogen (TN), and available phosphorus (AP), standard laboratory procedures were employed. The image analysis results for all of the years studied show that cultivated lands have increased in area at the expense of forest and grazing lands. Silt content, clay content, AP, and pH were significantly affected by land use as the main effect while the interaction effects of soil depth and land use were significant for total N and OM only. The highest (10.1 mg/kg) and the lowest (4.9 mg/kg) AP contents were observed in the forest and the grazing lands, respectively. Soil total N content was highest in the forest lands (0.32%) and lowest in cultivated lands (0.06%). Concerning OM content, the highest (11.0%) and the lowest (0.8%) values were recorded in the forest and cultivated lands, respectively. Generally, this study showed that land use changes have reduced the areal coverage by forest and grazing lands and have negatively affected the soil properties. This implies that land use change without soil fertility measures that are appropriate to the area could cause enhanced land degradation and thereby reduce the productivity of the study area soils.

## 1. Introduction

In Ethiopia, increased population pressure and related food needs are the basic drivers for the conversion of natural forests to grasslands and cultivated lands [1]. Changes in land use, mainly the conversion of natural forests to agricultural lands and settlements, are the most widely practiced activities in Ethiopia [2]. As a result, agricultural lands have been expanded at the expense of natural forests to meet the additional food demands for the increasing population [3]. Consequently, landscapes in various watersheds have been modified and the watershed capacity to produce important ecosystem services and functions is affected [4].

According to Desta and colleagues [5], the estimated annual harvesting rate of forest lands in the Amhara Regional State, for fuel, logging, and construction purposes reaches about 20,000 ha. Erosion is the major cause of the annual losses of over 1.5 billion tons of topsoil from the Ethiopian highlands, which could have added about 1.5 million tons of grains to the country's harvest [6]. The observed soil loss values have shown spatiotemporal variations and have ranged from the lowest 4.5 t. ha^−1^. yr^−1^ in the forest lands to the highest 65.9 t. ha^−1^. yr^−1^ in croplands [7]. This excessive removal of soils has led to the destruction of forest lands and resulted in a massive environmental degradation, a serious threat to sustainable agriculture.

Land use changes and the continuous use of lands for cultivation and grazing purposes resulted in a disastrous loss of soil nutrients, particularly in the highlands where erosion is more severe [2]. Such land use shifts are the causes of prevailing soil losses and degradation by enhancing the deterioration of the physical and chemical properties of soils. Soil compaction, loss of soil structure, soil OM degradation, soil acidity, and salinity are some examples of soil degradation problems due to unwise land use changes [8]. Undulating terrain, highly erosive rainfall, and inappropriate farming practices make soils highly vulnerable to erosion. Moreover, low fertilizer application rates, little turnover of crop residue, inadequate use of compost and farmyard manure, and poor land management practices have led to the depletion of soil nutrients [9].

Similarly, Gebrekidan and coworkers [10] also argued that changes in land use and soil management practices often modify most soil morphological, physical, chemical, and biological properties to the extent reflected in agricultural productivity. These authors reported an increment in bulk density, a deterioration of OM, and a reduction of cation exchange capacity (CEC), as the main impacts, which in turn reduce the fertility status of soils. In Ethiopia, several studies have been conducted to evaluate the effect of land use changes on soil properties [11,12]. On the other hand, some authors [13,14] tried to focus only on temporal LULC change studies without their implications on soils. However, in this study, we have attempted to integrate LULC dynamics over the years together with their cumulative effect on the current physical and chemical properties of the study area soils.

Most parts of the Estie District, in general, and the Tejibara watershed, in particular, have been gradually changing from the former forest land cover to the current mixed farming system. In the watershed, livestock production is as important as crop production, which forced farmers to allocate lands for intensive free grazing systems. Moreover, the increasing financial benefit from eucalyptus plantations served as an incentive for farmers to allot plots of lands for its cultivation. Considering the seriousness of the deteriorating soil property conditions, evaluating the LULC dynamics and studying the effects of different land use types on soil properties were found to be crucial to improve agricultural production and productivity on the one hand and to enhance sustainable land management systems on the other.

The study area is suitable for the production of different crops (such as barley, wheat, potato, teff, and maize), homestead tree plantations, and livestock rearing. However, the ever-increasing human population in the study area has dramatically reduced per capita landholding and created pressure on the available agricultural lands. This in turn has forced farmers to expand their farming activity on marginal lands such as steep lands and also to areas that are covered by vegetation. Moreover, cutting trees for fuelwood without replacement has become a serious problem contributing to LULC changes in the study area. Consequently, there is a belief that the abovementioned problems have brought a direct negative effect on the soil productivity in general and crop production and productivity in cultivated lands in particular. However, the extent and the rate of these problems in terms of land cover dynamics over the years and physical and chemical soil degradation were not systematically identified and properly quantified. Therefore, the major objective of this study is to evaluate the land cover dynamics and to determine the actual status of selected soil physicochemical properties in different land use types of the Tejibara watershed, central Amhara, Ethiopia.

## 2. Materials and Methods

### 2.1. Description of the Study Area

**Figure 1:**
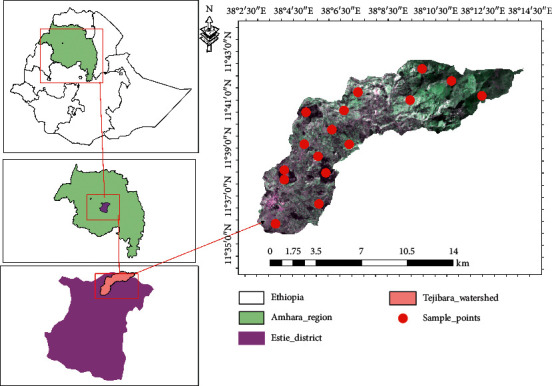
Location map of Tejibara watershed.

The study was conducted in the Tejibara watershed which is located in the Estie District, South Gondar Zone of the Amhara National Regional State (ANRS) at a distance of 55 km far from Debre Tabor, 106 km from Bahir Dar, and 668 km far from Addis Ababa. Tejibara watershed, with an estimated area of 10025.6 hectares, is located between geographic coordinates ranging from 11°37′10″–11°38′48″N and 38°3′15″–38°4′52″E (Figure 1).

Estie District includes three agroecological zones which are locally known as *Dega* (highland), *Weyna Dega* (midland), and *Kola* (lowland), from which the Tejibara watershed is located in the *Weyna Dega* agroecological zone. The study area is characterized by unimodal rainfall distribution pattern with about 1282 mm mean annual rainfall data, of which more than 77% of it occurs during the major rainy season (June to September). On the other hand, the annual mean minimum and maximum temperatures for the periods from 2004 to 2017 were 8.8 and 26.3°C, respectively.

The topography is related to relief characteristics and position on the landscape. Tejibara watershed has great geographic diversity ranging from flat plain to mountains in surface slope and low lands to highlands in altitude. The elevation of the watershed ranged from 2382 to 2660 meters above sea level (m.a.s.l.).

According to Estie District Agricultural Office (EDAO), the common soil types found in the Tejibara watershed are Nitisols and Vertisols [15]. However, the soil resource in the study watershed suffered from water erosion problem because of poor management of cultivated lands, overgrazing, and continuous trampling.

Trees and shrub species are mainly concentrated around the homesteads for better management and protection. The common tree species covering the forest land in the study area include Birbira (*Militia ferruginea*), Girar (*Acacia abyssinica*), Wanza (*Cordia africana*), Warka (*Ficus vasta*), Shola (*Ficus sycomorus*), *Eucalyptus globulus, Sesbania*, and other fodder tree and shrub species. Fodder tree and shrub species are important contributors to animal nutrition in the highlands areas. During the dry and crop-fallow season, farmers traditionally feed indigenous fodder species to meet the nutritional requirements of the grazing animals. *Eucalyptus globulus* is the main tree type planted in the area for construction and income generation purposes.

The farming practice is based on a mixed subsistence farming system which includes crop, forestry, and livestock activities with more emphasis on crop production. The study area is suitable for the growth of different major crops such as barley (*Hordeum vulgare*), wheat (*Triticum aestivum*), potato (*Solanum tuberosum*), teff (*Eragrostis tef*), and maize (*Zea mays*). These crops are growing under rainfed conditions and they are the means of livelihood for the farming community. On the other hand, cattle and small ruminants comprise the major livestock classes raised by the community in the watershed.

### 2.2. Methodology

#### 2.2.1. Remote Sensing Data Source

Multitemporal Landsat 5 Thematic Mapper (TM) imageries of 1989, 1999, and 2009 and Landsat 8 of OLI sensor image of 2019 with less than 10% cloud cover were used for mapping LULC classes of the Tejibara watershed (Table 1). The main application of both sensors (TM and OLI) is mapping and monitoring in the areas of forest, agriculture, coastal, inland water resources, and land use and land cover (LULC) changes. ASTER Digital Elevation Model (DEM) was also used to generate the watershed boundary.

#### 2.2.2. Land Use and Land Cover Classification

A detailed field survey was carried out throughout the watershed using Global Positioning System (GPS) instrument before the preprocessing and classification of satellite imagery. This survey was performed to obtain accurate locational point data for each LULC class included in the classification scheme as well as for the creation of training sites and signature generation.

Based on ground-truth and image analysis activities, five major LULC classes, viz., cultivated land, grazing land, forest land, bare land, and settlement were identified for mapping the entire watershed area (Table 2). Plantation lands, which were evaluated for physical and chemical properties of soils were not separately classified from forest lands due to close resemblance in spectral signatures. In supervised classification, spectral signatures are developed from specified locations in the image. Generally, a vector layer is digitized over the raster scene. The vector layer consists of various polygons overlaying different land use types. The training sites were used to develop spectral signatures for the outlined areas as described by Mallupattu and colleagues [16]. Pixel-based supervised image classification with maximum likelihood algorithm was employed to classify the images.

The land use and land cover maps of four different periods were used for postclassification comparison, which facilitated the estimation of changes in the land use category and dynamism with the changes. Postclassification comparison is the most commonly used quantitative method of change detection [17] with fairly good results. It involves independently produced spectral classification results from different datasets, followed by a pixel-by-pixel or segment-by-segment comparison to detect changes in the classes. Image analysis and LULC classification were processed through the application of ERDAS IMAGINE 2014 based on the similarities of their pixel reflectance values. A field survey was carried out to collect 180 sample points from all LULC classes for classification accuracy assessment. The ground-truth point's data and the classification results were compared and statistically analyzed using error matrices.

#### 2.2.3. Sampling Site Selection and Soil Sample Collection

To evaluate the influence of land use changes on soil physicochemical properties, soil samples were collected from four land use types, namely, cultivated land, grazing land, forest land, and plantation forest. To avoid the effect of topography on soil physicochemical properties, land use types with similar slope class (all soil samples were collected with slope ranges between 2 and 5% gentle sloping classes) were selected purposively before commencing the soil sample collection activities. From each land use type, composite soil samples were collected in triplicate from two soil depths (0–20 and 20–40 cm). Each sample was a composite of 10–15 subsamples collected in a crisscross (X) manner. In general, 24 soil samples were collected from four land use types and two soil depths (0–20 cm and 20–40 cm). Undisturbed soil samples were collected from the indicated two soil depths and four land use types to determine soil bulk density values. Cylindrical metal core with a volume of 100 cm^3^ was pressed into the soil until it is filled. The soil was trimmed at both ends with a knife and covered with a cap, labeled, and packed in a box.

#### 2.2.4. Soil Sample Preparation and Analysis

The collected composite soil samples were air-dried, mixed, crushed, and passed through a 2 mm sieve for all parameters studied except for total nitrogen (TN) and organic carbon (OC), which was then passed through a 0.5 mm sieve. The soil physical and chemical analyses were carried out in Gondar and Bahir Dar (Amhara Design and Supervision Work Enterprise) soil testing laboratories.

Bulk density (BD) of soils was estimated from undisturbed soil samples collected from each land use type using a core sampler and weighed at field moisture content and then dried in an oven at 105°C for 24 hours. Bulk density values were later calculated using the following equation:

(1) $$\begin{array}{c} \text{BD}=\frac{\text{DM}}{V}, \end{array}$$

where BD is the bulk density (g/cm^3^), DM is the dry mass (g) of the soil sample, and *V* is the volume of core sampler (cm^3^).

The hydrometer method was used to determine soil particle size distribution. Finally, soil textural names were determined following the textural triangle of the USDA system as described by Rowell [18].

Soil organic carbon (SOC) was determined by Walkley–Black rapid titration method as described by Sarkar and Haldar [19], and soil organic matter (SOM) was calculated by multiplying SOC values with a factor of 1.724. Soil pH was determined potentiometrically in a 1 : 2.5 soil-to-water suspension using a pH meter. Total nitrogen was determined using the micro-Kjeldahl digestion, distillation, and titration procedure as described by Bremner and Mulvaney [20]. Available phosphorus was measured following the Olsen method [21]. To determine the cation exchange capacity (CEC), the soil samples were first leached with 1 M ammonium acetate and washed with ethanol and the adsorbed ammonium was replaced by Na. Then, the CEC was measured titrimetrically by distillation of the ammonia that was displaced by sodium [22]. Exchangeable K^+^ was then analyzed by using a flame photometer after extracting the soil samples by ammonium acetate (1N NH_4_OAc) at pH 7.0 [18].

#### 2.2.5. Statistical Analysis

Statistical differences in soil characteristics among land use types and soil depths were tested using a two-way analysis of variance (ANOVA) following the General Linear Model (GLM) procedure of Statistical Analysis System (SAS) version 9.4. For means with significant (*P* < 0.05) differences, mean comparison was performed using the least significant difference (LSD at 5% level of significance) tests.

## 3. Results

### 3.1. Accuracy Assessment and Observed LULC Changes in the Study Watershed

**Figure 2:**
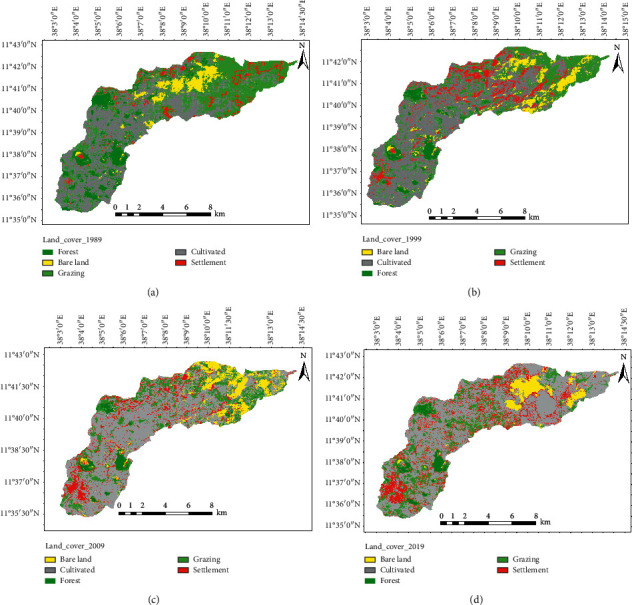
Land use and land cover in (a) 1989, (b) 1999, (c) 2009, and (d) 2019.

The overall accuracy assessment and kappa coefficient of LULC classification were 90 and 87%, respectively (Table 3). This indicated the presence of strong agreement between the classified image and the reference data. The largest portion of the watershed is occupied by cultivated land while the smallest is covered by forest in the final year of analysis. The forest and grazing lands have been reduced by 7.7 and 17.8%, respectively, as compared with the base year. In contrast, the size of the cultivated land and settlement increased by 15.9 and 9.9% as compared to the reference year of 1989 (Figure 2 and Table 4). Most of the lands in the study area, which were previously covered by forests and grazing lands, are now converted to cultivated lands (Figure 2 and Table 4). For example, the estimated 10.9% forest coverage in 1989 was dropped down to 3.2% in 2019 while the cultivated land showed an increasing rate from its 40.4% area coverage in the year 1989 to 56.3% in 2019.

### 3.2. Physical Properties of Soils under Different Land Uses

The analysis results revealed that sand particle size values were different for different land use types and ranged from the lowest 33.4% in eucalyptus plantation to the highest 45.8% in grazing lands. However, the observed numerical differences were not significant (*P* > 0.05) when the sand contents are evaluated in statistical terms. The sand particle size value in the eucalyptus plantation was the lowest as compared to other land use types (Table 5). Silt particle size values were also different for different land use types and ranged from the lowest 33.6% in grazing land to the highest 40.3% in cultivated land (Table 5). The silt particle size value of grazing land was significantly (*P* < 0.05) lower than the remaining three land use types. Clay contents were significantly (*P* < 0.01) different for different land use types (Table 5). The value of clay content was inversely related to sand fraction. It ranged from the lowest 15.3% in forest land to the highest 31.0% in the eucalyptus plantation.

In terms of textural classes, soil samples collected from natural forest, grazing land, and cultivated lands were classified as loam soil while soils from eucalyptus plantation were classified as clay loam (Table 5). However, this clay loam-rich eucalyptus plantation has been continually affected by problems of inadequate aeration, waterlogging, increased runoff and erosion, and the problem of workability during very dry and very wet periods of the year (field observation).

The bulk density values of the analyzed soils were highly significantly (*P* ≤ 0.001) affected by the main effects of land use types and soil depths. The mean soil bulk density values across land use types were 1.5 g/cm^3^, 1.4 g/cm^3^, 1.4 g/cm^3^, and 1.0 g/cm^3^ in the cultivated land, eucalyptus plantation, grazing, and forest lands, respectively (Table 5). Considering the soil depths, the bulk density values were significantly (*P* ≤ 0.05) increased from the upper 0–20 cm depth (with a mean bulk density value of 1.2 g/cm^3^) to the lower 20–40 cm depth (with a mean bulk density value of 1.4 g/cm^3^) in almost all land use types. On the other hand, soil bulk density values were not significantly (*P* > 0.05) affected by the interactions of land use types and soil depths (Table 6). However, the soil bulk density values were numerically different with the lowest values of 0.9 and 1.1 g/cm^3^ in the surface and subsurface layers of forest land, respectively. However, the highest soil bulk density values were observed in the surface and subsurface soils of cultivated lands (1.4 and 1.6 g/cm^3^, respectively). Soil moisture content was significantly (*P* ≤ 0.05) affected by land use types (Tables 5 and 6). In this study, forest soils were found to contain significantly higher soil moisture content (25.5%), followed by cultivated land (23.7%), eucalyptus plantation (19.2%), and grazing land (17.7%).

### 3.3. Soil Chemical Properties under Different Land Use Types

Soil pH showed a highly significant difference (*P* ≤ 0.01) among the land use types (Table 7). The lowest pH (6.0) was observed in soil samples collected from eucalyptus plantation whereas comparatively higher (6.5) soil pH was observed in samples collected from cultivated and forest land use types (Table 8). Electrical conductivity (EC) of soils was highly significantly affected (*P* ≤ 0.01) by land use, soil depth, and their interaction (*P* ≤ 0.05) (Table 7). Considering the main effects of land use types, the highest (0.10 ms/cm) and the lowest (0.02 ms/cm) EC values were obtained under the forest and the croplands, respectively (Table 8). Concerning the interaction effect of land use by soil depth, the highest interaction mean value of EC (0.14 ms/cm) was obtained in the surface soil (0–20 cm) layer of the forest land, whereas the lowest value was observed in both the surface and subsoil layers of the cultivated land (Table 8). Since EC values are negligible (they are generally less than 0.1 ms/cm), the probability of the formation of actual and potential salinity problems in the study area is unlikely.

Organic matter content was highly significantly (*P* ≤ 0.01) affected by land use, soil depth, and the interaction of land use by soil depth (Tables 7–9). Consequently, soil OM content was highest (11.0%) under the forest land and lowest (0.83%) in the cultivated lands. Considering the two soil depths, higher average OM content was observed in the surface (5.6%) than subsurface (3.8%) layers (Table 8). Considering the interaction effect of land use by soil depth, the highest (12.9%) and the lowest (0.6%) values of OM contents were recorded at the surface (0–20 cm) layer of the forest and the subsurface (20–40 cm) layer of the cultivated lands, respectively (Table 8). According to Tekalign [23], the levels of OM are classified as very low (<0.9%), low (0.9–2.6%), medium (2.6–5.2%), and high (>5.2%). Therefore, the OM content of soils in the study area was recorded as high (11.0%), moderate (4.4%), low (2.5%), and very low (0.8%) for the natural forest, eucalyptus plantation, grazing land, and cultivated lands, respectively.

The total N content of soils was highly significantly (*P* ≤ 0.01) affected by land use, soil depth, and the interaction of land use by soil depth (Tables 7–9). The average values of total N in the study area ranged from the highest 0.32% in the forest lands to the lowest 0.06% in cultivated lands. As per Tekalign [23] ratings, high (0.32%) total N content was observed in the forest land, moderate (0.18%) in eucalyptus plantation, and low (0.11% and 0.06%) in grazing and cultivated lands, respectively. The mean total N content decreased considerably from 0.18% in the surface (0–20 cm) to 0.15% in the subsurface (20–40 cm) soil layers (Table 8). Similarly, total N was highly significantly affected by the interaction of land use by soil depth and the highest (0.35%) value of total N was recorded at the surface (0–20 cm) layer of the forest land (Table 9). On the other hand, the lowest (0.05%) interaction mean value of total N was observed in the surface (0–20 cm) layer of the cultivated land.

The available phosphorus (AP) content was significantly (*P* ≤ 0.05) affected by land use types. However, soil depth and its interaction with land use did not show significant variation (Tables 7–9). The contents of AP in the forest and cultivated lands appeared to be significantly higher than the plantation and grazing land use types. Accordingly, comparatively highest (10.1 mg/kg) and the lowest (4.9 mg/kg) AP contents were observed under the forest and the grazing lands, respectively (Table 8). The data also revealed that AP was higher (7.5 mg/kg) in the surface soil (0–20 cm) than in the subsurface soil (20–40 cm) layer (6.8 mg/kg).

Exchangeable K content was not significantly affected by land use, soil depth, and the interaction of land use by soil depth (Tables 7 and 8). Indeed, there was a numerical difference among land use types and recorded as 2.3 cmol_c_/kg, 1.8 cmol_c_/kg, 1.5 cmol_c_/kg, and 1.2 cmol_c_/kg in the forest, eucalyptus plantation, cultivated, and grazing lands, respectively. The CEC values of the soils in the study area were not significantly affected by land use, soil depth, and the interaction effects of land use by soil depth though there are some numerical variations among land use types (Tables 7 and 8). The CEC values of different land uses in the study area are found under the high category.

## 4. Discussion

### 4.1. Land Use and Land Cover Dynamics

The LULC dynamics showed that the forest and grazing lands have been reducing consistently for the three decades due to the demand for cultivated lands and settlement by the ever-increasing population residing in the watershed. Trees in the watershed have been cleared for wood, charcoal, house construction, and household furniture. Farmers start to settle and cultivate crops on these lands legally and illegally. Since there is no practically implemented land use policy in the area, grazing and forest lands were illegally converted to agriculture and settlement lands. Similarly, in different parts of Ethiopia, several authors reported a decrease in forest cover and an increase in the area of cultivated lands. For example, Fasika and colleagues [24] indicated that the proportion of a study area covered by forest and agriculture decreased by 60.6 ha (12.7%) within 32 years taken as a study period (between 1985 and 2017). Tefera and Sterk [25] reported that croplands were endlessly expanding from comparatively flat areas in 1957 and 1980 to steep lands in 2001 at the expense of grazing lands, which are found in Fincha watershed, western highlands of Ethiopia. Markos and colleagues [26] showed that cultivated lands and settlement land expanded by 67.4 and 532%, respectively, whereas forest land and shrubland and grassland declined by 66.4 and 18.4%, respectively, over the analysis period. Expansion of agriculture in Ethiopia is related to a change in land use policy with a change of regimes from Derg to Ethiopian People Revolutionary Democratic Front [27]. On top of that, due to an increase in rural population pressure and related food demands, farmers residing in the watershed were forced to plow lands that have been covered with forests and grasses [28]. Such unplanned land use changes have led to the removal of fertile soils through erosion and the development of gullies in the watershed. Removal of surface vegetation decreases infiltration in the soil and results in increased runoff. Several researchers suggested that the high rate of soil erosion and productivity decline in Ethiopia is related to deforestation and LULC changes.

### 4.2. Soil Physical Properties under Different Land Uses

The relative variation in sand fraction could be related to distractions of soil structure by animal trampling due to free grazing that causes fine soil particles more vulnerable to water and wind erosion, and sand fraction remained behind fine particles. This result is in agreement with the findings by Mulugeta [29] and Kiflu and Sheleme [30] who reported that sand fraction is greater in grazing lands as compared to other land use types. On the other hand, contradicting results were also reported by some other authors indicating the highest sand fraction content in the forest lands as compared to other land use types [31,32]. The lowest content of clay fraction of forest land may be attributed to downward translocation of clay particles from the upper mineral horizons of the forest land since eluviation is most active in forest lands [33].

Even though the texture is an inherent soil property, management practices may contribute indirectly to the changes in particle size distribution particularly in the surface layers as a result of removal of soil by sheet and rill erosions, and mixing up of the surface and the subsurface layers during continuous tillage activities. Therefore, differences in particle size distribution were observed, which can be attributed to the impact of deforestation and farming practices such as continuous tillage or cultivation and intensive grazing. The loam textural classes of natural forest, grazing, and cultivated lands are appropriate for plant growth because these soils are permeable to air, water, and plant roots and storehouses for plant nutrients as compared to clay loam textural classes [34].

The highest soil bulk density observed in cultivated lands could be related to soil compaction as a result of frequent trampling of draft animals, commonly oxen, and farm implements during the wet season, and depletions of OM compared to natural forest lands. Relatively higher biomass production in the forest land causes continuous litter addition to the soil that makes the soil to have a lower bulk density as compared to crop and grazing lands. Similar results were reported by some authors [31, 32, 35, 36] who stated lower soil bulk density values in the forest land as compared to cultivated and grazing lands.

Bulk density typically increases with soil depth since subsurface layers are more compacted and have lower OM content, less aggregation, less root penetration, and limited pore spaces as compared to surface layers. In the same way, different researchers have indicated the increase in bulk density values as one moves from the surface to subsurface soils [37]. Increased bulk density could reduce soil porosity and thereby affects soil water and air movements in the study area soils. Moreover, higher soil bulk density conditions could restrict plant root developments and subsequent agricultural productivity. A similar finding was reported by Gebrelibanos and Mohammed [38].

Among different land uses studied, forest lands were found to have higher SMC values. This could be related to the high water-holding capacity of the forest soil as a result of comparatively higher (11.0%) OM content (Table 8), well-structured soils, and less evaporation loss because of shading effect. This result is in agreement with the study reported by Negassa [39] who reported that the water content at the permanent wilting point was highest (19.7%) under the forest land than it was in cultivated lands (16.6%) and grazing lands (16.2%). Water infiltration in undisturbed forest soils is enhanced by both preferential flows along with tree roots and accumulation of absorbent humus on the soil surface, thereby significantly reducing the volume, velocity, and erosive and leaching capacity of surface runoff [40].

### 4.3. Soil Chemical Properties under Different Land Uses

Soil pH ranged from moderately acidic (for eucalyptus plantation) to slightly acidic (for the remaining land use types) reactions [23]. Land use changes from natural forest to other land uses resulted in a reduction of soil pH which is in line with the study reported by Selassie and colleagues [35]. The lowest pH value observed under the eucalyptus plantation might be due to a higher rate of microbial oxidation of organic materials that produce organic acids, which provide H^+^ ions to the soil solution, thereby lowering soil pH. Concerning soil depths, the soil pH in the watershed increased with increasing soil depth, and it agrees with the study reported by Habtamu and coworkers [31].

Though soil OM content was variable for different land use types and soil depths studied, the highest value was recorded in the natural forests. In this study, OM content showed a decreasing trend with increasing soil depths across all land use types. This could be related to the applications of organic inputs and the highest microbial activities on the surface than subsurface soils. The result is in agreement with the findings of different authors [34, 35] who reported that the lowest OM was observed in cultivated lands and highest in the forest lands. The authors further tried to relate poor OM contents in cultivated lands to the complete removal of biomass from the fields, severe deforestation, intensive cultivation, steep relief conditions, and to the subsequent erosion hazards.

The very low value of soil total N observed under cultivated lands may be due to continuous cultivation, soil erosion, plant uptake, and volatilization of N resulting from increased oxidation of nitrogenous compounds. Similarly, low carbon input obtained from the subsistence agricultural crop production system could not compensate N losses by OM mineralization, leaching, and denitrification. On the other hand, the relatively high value of total N observed under forest lands may be due to high OM accumulation and to good forest microclimate conditions which might have moderated the soil temperature and thereby decreased total N loss by volatilization. According to Walworth [41], the OM in the soil contributes roughly 95% for soil total N contents. The result is consistent with the observation made in central Ethiopia [42].

According to AP rating suggested by Cottenie [43], all land use types are found to contain very low to low (<5 and 5–9 mg/kg, respectively) AP values except forest lands, which contain medium (10–17 mg/kg) AP values. The low soil AP contents observed in the watershed are in agreement with the results reported by Fekadu and colleagues [44], who reported that the availability of P in most Ethiopian soils has been declining due to fixation, crop removal, and erosion. Weathered soil minerals, organic fertilizer, and inorganic fertilizer are important pools of soil P [45]. Thus, the fact that soils in the forest lands have higher AP than the other land use types could be attributed to P return through litterfall to soil surface [46, 47].

The exchangeable K values observed in this study were above the critical levels of 0.4 cmol_c_/kg for the production of most crop plants [48], so the results indicate that exchangeable K concentration is not limiting in the soils of the study area. Relatively, higher CEC in forest lands might be related to the accumulations of higher OM. On the other hand, the observed comparatively lower CEC values under cultivated lands might be attributed to low basic cations due to leaching, and soil erosion. In some other studies, the depletion of exchangeable bases as a result of intensive cultivation and application of inorganic fertilizer was mentioned as a reason for a decrease in CEC values under cultivated lands [49].

## 5. Conclusions

The analysis results showed that unwise LULC changes were the prime factors responsible for the widespread expansion of cultivated lands at the expense of the forest and grass covers. The ever-increasing population pressure observed in the years 1989–2019 and its related demand for agricultural lands has negatively affected the existing LULC of forest and grazing lands. These changes were also accompanied by changes in soil physical and chemical properties. Relatively higher and lower BD values were recorded in the respective cultivated and forest lands, mainly as a result of soil compaction and OM accumulation. The presence of relatively higher CEC in forest lands as compared to other land uses might be related to the accumulations of higher OM. The higher and the lower AP contents were observed under the forest and the grazing lands as a result of P return through litterfall and removals of P due to overgrazing in grazing lands, respectively. Currently, there is an increased agricultural production need to meet the food demand of the growing population. Hence, it is more likely that the change of natural forest lands to other land uses will continue further. This result implies that land use changes without appropriate to the area soil fertility improvement measures would aggravate the existing soil degradation problems and would create an ecological imbalance in the study area. Hence, the use of the available land resources in a planned manner, improving the management of soil resources, and making them fit for sustainable agricultural use would be the most useful strategies to protect biological diversity from agricultural land expansion.

## Figures and Tables

**Table 1:** Description of satellite images.

Satellite	Sensor	Date of acquisition	Pixel resolution (m)	No. of bands
Landsat 5	TM	03/10/1989	30	7
Landsat 5	TM	16/11/1999	30	7
Landsat 5	TM	11/11/2009	30	7
Landsat 8	OLI	25/12/2019	30	11

**Table 2:** Description of land use and land cover classes.

Land use/land cover classes	Descriptions
Cultivated land	Areas allotted to rainfed and irrigated cultivation, including fallow plots, cultivated land mixed with some bushes, trees, and the scattered rural settlements included within the cultivated fields
Forest land	Areas covered by trees forming closed or nearly closed canopies, forest, plantation forest, dense (50–80% crown cover)
Grazing land	Areas of land where small grasses are the predominant natural vegetation usually used for grazing
Bare land	Areas with little or no “green” vegetation present due to erosion, overgrazing, and crop cultivation
Settlement	Areas covered with buildings in rural and urban. It includes commercial, residential, industrial, and transportation infrastructures

**Table 3:** Accuracy assessment matrix for LULC.

Classification class	Reference class						
	Forest	Grass	Cultivated	Bare land	Settlement	Row total	User' accuracy (%)
Forest	**23**	0	0	0	0	23	100.0
Grass	0	**36**	6	0	0	42	85.7
Cultivated	0	3	**56**	0	2	61	91.8
Bare land	0	2	0	**24**	2	28	85.7
Settlement	0	0	1	2	**23**	26	88.5
Column total	23	41	63	26	27	180	
Producers' accuracy (%)	100	87.8	88.9	92.3	85.2		
Overall accuracy	90%						
Kappa coefficient	87%						

**Table 4:** Land use and land cover (LULC) change from 1989 to 2019.

Land cover type	LULC 1989		LULC 1999		LULC 2009		LULC 2019		Change in % (1989–2019)
	Area (ha)	% of area	Area (ha)	% of area	Area (ha)	% of area	Area (ha)	% of area	
Forest	1096.3	10.9	616.5	6.1	367.2	3.7	321.2	3.2	−7.7
Cultivated	4054.1	40.4	5067.5	50.6	5566.1	55.5	5646.8	56.3	15.9
Grazing	3719.2	37.1	2577.2	25.7	1967.6	19.6	1937.3	19.3	−17.8
Bare land	671.0	6.7	754.5	7.5	1023.6	10.2	635.3	6.4	−0.3
Settlement	485.0	4.8	1009.9	10.1	1101.2	11.0	1484.9	14.8	10

Total	10025.6	100.0	10025.6	100.0	10025.6	100.0	10025.6	100.0	

**Table 5:** Main effects of land use and soil depth on selected physical properties of the soils.

Land use type	Sand (%)	Silt (%)	Clay (%)	STC	BD (g/cm^3^)	SMC
Natural forest	45.1^a^	39.6^a^	15.3^c^	Loam	1.0^c^	25.5^a^
Eucalyptus plantation	33.4^b^	35.6^ab^	31.0^a^	Clay loam	1.4^b^	19.2^bc^
Grazing	45.8^a^	33.6^b^	20.6^bc^	Loam	1.4^b^	17.7^c^
Cultivated	37.1^ab^	40.3^a^	22.6^b^	Loam	1.5^a^	23.6^ab^
LSD (0.05)	11.4	5.1	7.3		0.1	5.4
CV (%)	22.7	10.9	26.3		8.4	20.3

*Soil depth*						
0–20 cm	43.1	36.6	20.3	Loam	1.2^b^	19.9
20–40 cm	37.6	37.9	24.5	Loam	1.4^c^	23.2
LSD (0.05)	NS	NS	NS		0.10	NS
CV (%)	22.7	10.9	26.3		8.4	20.3

Main effect means within a column followed by the same letter are not significantly different from each other at *P* ≤ 0.05. CV = coefficient of variation; LSD = least significant difference; NS = not significant; STC = soil textural class; BD = bulk density; SMC = soil moisture content.

**Table 6:** Mean square (MS) and results of two-way analysis of variance of soil physical properties.

Physical properties	Land use			Soil depth (cm)			Interaction effects		
	MS	F	P	MS	F	P	MS	F	P
Sand (%)	220.61	2.61	0.092^ns^	181.5	2.15	0.164^ns^	17.5	0.21	0.889^ns^
Silt (%)	61.33	3.65	0.039^*∗*^	10.67	0.64	0.438^ns^	25.77	1.54	0.249^ns^
Clay (%)	253.94	7.32	0.004^*∗*^	104.17	3.00	0.105^ns^	25.94	0.75	0.541^ns^
BD (g/cm^3^)	0.269	21.93	0.0001^*∗∗*^	0.096	7.84	0.014^*∗*^	0.015	1.21	0.342^ns^
SMC (%)	80.87	4.23	0.0252^*∗*^	64.059	3.35	0.089^ns^	56.21	2.94	0.069^ns^

^*∗*^Significant at *P* ≤ 0.05; ^*∗∗*^significant at *P* ≤ 0.01; ns = not significant; MS = mean square; F = calculated value; P = probability; BD = bulk density.

**Table 7:** Mean square (MS) and results of two-way analysis of variance of soil chemical properties.

Chemical properties	Land use			Soil depth (cm)			Interaction effects		
	MS	F	P	MS	F	P	MS	F	P
pH (H_2_O)	0.348	11.71	0.0004^*∗∗*^	0.0247	0.83	0.3774^ns^	0.0366	1.23	0.3356^ns^
EC (dS/m)	0.0074	62.66	0.0001^*∗∗*^	0.0043	36.02	0.0001^*∗∗*^	0.0011	9.38	0.0012^*∗∗*^
OM (%)	118.6	175.21	0.0001^*∗∗*^	20.075	29.66	0.0001^*∗∗*^	4.615	7.33	0.0034^*∗∗*^
TN (%)	0.075	80.21	0.0001^*∗∗*^	0.0045	4.88	0.0444^*∗*^	0.0036	3.85	0.0336^*∗*^
AP (mg/kg)	3114.72	4.00	0.0298^*∗*^	363.015	0.47	0.5056^ns^	1786.79	2.3	0.1222^ns^
CEC (cmol_c_/kg)	12.409	2.82	0.0776^ns^	0.1998	0.05	0.8345^ns^	3.7954	0.86	0.4841^ns^
K (cmol_c_/kg)	0.953	3.04	0.064^ns^	0.011	0.03	0.8551^ns^	0.542	1.73	0.2064^ns^

^*∗*^Significant at *P* ≤ 0.05; ^*∗∗*^significant at *P* ≤ 0.01; ns = not significant; MS = mean square; F = calculated value; P = probability; EC = electrical conductivity; OM = organic matter; TN = total nitrogen; AP = available phosphorus; CEC = cation exchange capacity.

**Table 8:** Main effects of land use and soil depth on some chemical properties of the soils.

Land use types	pH (H_2_O)	EC (ms/cm)	OM (%)	TN (%)	Av. P (mg/kg)	Ex. K (cmol_c_/kg)	CEC (cmol_c_/kg)
Natural forest	6.5^a^	0.10^a^	11.0^a^	0.32^a^	10.1^a^	2.2	40.4
Eucalyptus plantation	6.0^b^	0.06^b^	4.4^b^	0.18^b^	5.9^b^	1.8	39.7
Grazing	6.4^a^	0.04^c^	2.5^c^	0.11^c^	4.9^b^	1.2	37.2
Cultivated	6.5^a^	0.02^d^	0.8^d^	0.06^d^	7.6^ab^	1.5	38.1
LSD (0.05)	0.21	0.01	1.02	0.04	3.45	NS	2.60
CV (%)	2.7	19.2	17.5	18.2	38.9	33.6	5.4

*Soil depth*							
0–20 cm	6.3	0.07^a^	5.6^a^	0.18^a^	7.5	1.7	38.9
20–40 cm	6.4	0.04^b^	3.8^b^	0.15^b^	6.8	1.6	38.8
LSD (0.05)	NS	0.01	0.72	0.03	NS	NS	NS
CV (%)	2.7	19.2	17.5	18.2	38.9	33.6	5.4

The main effect means within a column followed by the same letter are not significantly different from each other at *P* ≤ 0.05. NS = not significant; CV = coefficient of variation; LSD = least significant difference.

**Table 9:** Interaction effects of land use and soil depth on selected soil chemical properties.

Land use types	pH (H_2_O)		EC (ms/cm)		OM (%)		Total N (%)		Av. P(mg/kg)		CEC(cmol_c_/kg)		Ex. K(cmol_c_/kg)	
	Soil depth (cm)													
	0–20	20–40	0–20	20–40	0–20	20–40	0–20	20–40	0–20	20–40	0–20	20–40	0–20	20–40
Forest land	6.5	6.5	0.14^a^	0.07^b^	12.9^a^	9.1^b^	0.35a	0.29^b^	10.9	9.3	40.9	39.8	2.03	2.3
Eucalyptus plantation	6.0	5.9	0.07^b^	0.05^c^	5.9^c^	2.9^d^	0.22c	0.14^d^	7.9	3.9	40.7	38.8	2.19	1.3
Grazing land	6.3	6.5	0.05^c^	0.04^cd^	2.7^d^	2.4^de^	0.11de	0.11^de^	2.8	7.0	36.8	37.7	1.31	1.2
Cultivated land	6.4	6.6	0.02^de^	0.02^e^	1.0^ef^	0.6^f^	0.05f	0.07^ef^	8.5	6.7	37.4	38.8	1.23	1.7
LSD (0.05)	NS		0.02		1.44		0.053		NS		NS		NS	
CV (%)	2.7		19.2		17.5		18.2		38.9		5.4		33.6	

^*∗*^Interaction means within a specific soil parameter followed by the same letter are not significantly different from each other at *P* ≤ 0.05. CV = coefficient of variation; LSD = least significant difference.

## Data Availability

The data used to support the findings of this study are available on request.
